# Prefrontal cortex and executive function in young children: a review of NIRS studies

**DOI:** 10.3389/fnhum.2013.00867

**Published:** 2013-12-17

**Authors:** Yusuke Moriguchi, Kazuo Hiraki

**Affiliations:** ^1^Department of School Education, Joetsu University of EducationJoetsu, Japan; ^2^Japan Science and Technology Agency, PRESTOTokyo, Japan; ^3^Department of Systems Science, University of TokyoTokyo, Japan; ^4^Japan Science and Technology Agency, CRESTOTokyo, Japan

**Keywords:** prefrontal cortex, executive function, young children, NIRS, developmental disorders

## Abstract

Executive function (EF) refers to the higher-order cognitive control process for the attainment of a specific goal. There are several subcomponents of EF, such as inhibition, cognitive shifting, and working memory. Extensive neuroimaging research in adults has revealed that the lateral prefrontal cortex plays an important role in EF. Developmental studies have reported behavioral evidence showing that EF changes significantly during preschool years. However, the neural mechanism of EF in young children is still unclear. This article reviews recent near-infrared spectroscopy (NIRS) research that examined the relationship between the development of EF and the lateral prefrontal cortex. Specifically, this review focuses on inhibitory control, cognitive shifting, and working memory in young children. Research has consistently shown significant prefrontal activation during tasks in typically developed children, but this activation may be abnormal in children with developmental disorders. Finally, methodological issues and future directions are discussed.

Executive function (EF) refers to the higher-order cognitive control process for the attainment of a specific goal. EF plays an important role in multiple areas of child development such as social cognition, communicative behavior, and moral behavior (Kochanska et al., 1997; Carlson and Moses, 2001; Moriguchi et al., 2008, 2010a). It has been shown that EF has several subcomponents in adults and older children (Miyake et al., 2000; Lehto et al., 2003; Huizinga et al., 2006). Miyake et al. (2000) have shown that the three major components of EF, namely, inhibition, shifting, and updating (working memory), are separable even though they are moderately correlated. However, there are still controversies regarding the data for preschool-aged children. Theoretically, there might be three components of EF in young children (Garon et al., 2008), but, empirically, a single-factor model (general EF) has been sufficient to account for the data in preschool-aged children (Wiebe et al., 2008).

## Behavioral and anatomical evidence

Extensive research has shown that cognitive shifting rapidly develops during preschool years. One task widely used in such research is the Dimensional Change Card Sort (DCCS) task (Zelazo et al., 1996; Kirkham et al., 2003; Moriguchi et al., 2010b). In this task, children are asked to sort cards that have two dimensions, such as color and shape (e.g., red boats, blue rabbits). There are two phases to the task. During the preswitch phase, children are asked to sort cards according to one dimension (e.g., color) for several trials. During the postswitch phase, children are asked to sort the cards according to the other dimension (e.g., shape) for several trials. It has been repeatedly shown that most 3-year-olds correctly perform the preswitch phase, but show difficulty with the postswitch phase (Zelazo et al., 1996; Moriguchi et al., 2012). Four- and five-year-old children correctly sort the cards according to the second dimension. The DCCS is used to index cognitive shifting as well as EF in general (Garon et al., 2008).

Researchers have used the Stroop-like Day-Night task and the Black-White task to examine the development of inhibitory control in young children (Gerstadt et al., 1994; Simpson and Riggs, 2005; Moriguchi, 2012). In the Day-Night task, children are instructed to say “day” in response to a picture of a moon with some stars and “night” in response to a picture of a sun. In order to perform the task correctly, children have to inhibit the dominant response (e.g., children have to inhibit day responses when presented with a sun card). For this task, response accuracy and latency has been shown to improve between 3 and 5 years of age. Although it has been suggested that both inhibition skills and working memory are needed to pass the task, this task is often used as an index of inhibition skills (Carlson and Moses, 2001).

The development of working memory is measured by the self-ordered searching task (Luciana and Nelson, 1998). In this task, several colored squares are presented on a computer screen and each square contains a token. The children’s task is to touch and open the squares and find as many tokens as possible. Each square has only one token; therefore, children must keep the previously selected squares in mind and use this information to inform subsequent responses. Luciana and Nelson (1998) gave 4- to 8-year-old children the task and found that the performance improved during this period. Specifically, 4-year-old children performed worse in three- and four-item searches than older children. In the six-item search, 7- and 8-year-old children outperformed younger children. Previous research has consistently shown that children develop visuospatial working memory during their preschool years (Garon et al., 2008).

Although behavioral evidence is accumulating, the neural basis of EF in young children is still unknown. There is some anatomical evidence that the prefrontal cortex develops during preschool years. Recent structural magnetic resonance imaging (MRI) studies have shown changes in brain structure over time within an individual. Studies by Giedd and colleagues (Giedd et al., 1999; Gogtay et al., 2004) have indicated that gray matter in the prefrontal cortex shows inverted U-shape changes during childhood. The volume in the prefrontal regions increase with age until adolescence (Tanaka et al., 2012). Additionally, Giedd et al. (1999) have shown that white matter volume in the frontal area increases linearly during the ages of 4–20 years. This evidence suggests the likelihood of structural changes within the prefrontal cortex during preschool years.

## Neural basis of cognitive shifting and inhibitory control in young children

Little neuroimaging data have demonstrated the functional development of the prefrontal cortex during preschool years. However, recently, studies using near-infrared spectroscopy (NIRS) have shown that prefrontal activation is developmentally correlated with EF in young children. With NIRS, it is possible to monitor cerebral hemodynamics by measuring changes in the attenuation of near-infrared light passing through the tissue. Because NIRS is noninvasive and does not require fixing of the body as in functional MRI (fMRI), it is often used for brain imaging studies in infants and children. There are three NIRS parameters: oxygenated hemoglobin level, deoxygenated hemoglobin level, and total hemoglobin level; however, this review mainly concentrate on oxygenated hemoglobin findings because most of the previous research on young children consistently reported the oxygenated hemoglobin as an index of brain activation. The change in oxygenated hemoglobin level is considered to be a good indicator of brain activity (Strangman et al., 2002; but see also Huppert et al., 2006).

The spatial and depth sensitivity to activations in a region in NIRS system is limited compared to other neuroimaging technique such as fMRI (Strangman et al., 2013). Nevertheless, some NIRS researches are based on the previous other neuroimaging research such as fMRI to decide region of interest. Brain imaging studies using fMRI have shown that adult participants recruit inferior and dorsolateral prefrontal regions during cognitive shifting tasks, such as the Wisconsin Card Sorting Test (WCST; Konishi et al., 1998; Monchi et al., 2001). In the WCST, participants are asked to sort cards depicting geometric features, such as shape, color, and number according to rules that are detected through feedback given by an experimenter. After the participants figure out the rule and sort the cards for several trials, the rule suddenly changes and participants must adjust to the rule change depending on the feedback. In this task, participants recruit prefrontal areas, as well as the parietal cortex, when they have to switch from one rule to another (Konishi et al., 1998; Monchi et al., 2001).

**Figure 1:**
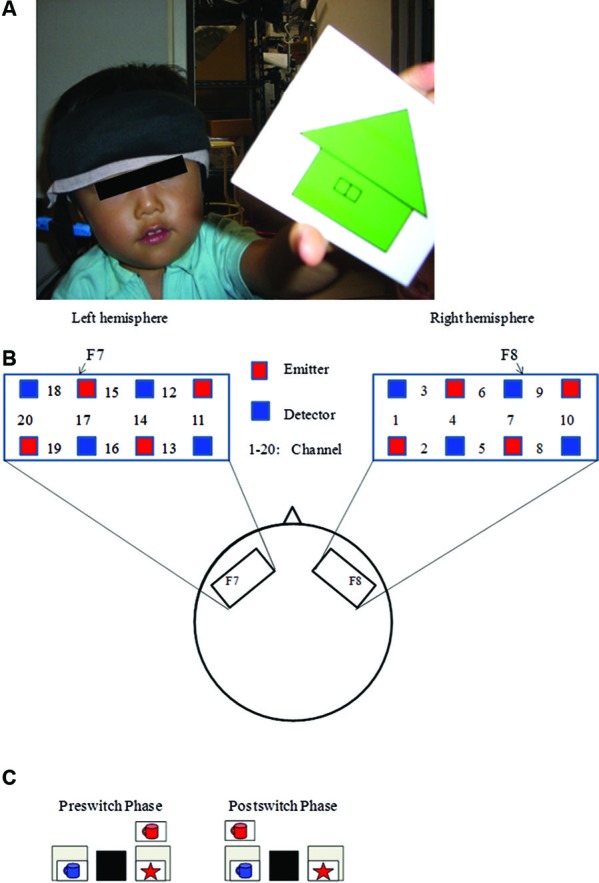
**Experimental settings**. **(A)** A child with NIRS probe. **(B)** The NIRS probe was attached to the inferior prefrontal area. Each channel consisted of one emitter optode and one detector optode. The region of interest was located near F7/8, which corresponds to ch 15, 17, 18 and 6, 7, 9, respectively. **(C)** An example of preswitch and postswitch phases in the DCCS task. Figure from Moriguchi and Hiraki (2009). Neural origin of cognitive shifting in young children. *Proceedings of the National Academy of Sciences of the United States of America*, 106, 6017–6021.

Recently, Moriguchi and Hiraki (2009) examined the neural basis of cognitive shifting in young children (Figure 1). In this cross-sectional study, 3-year-old children, 5-year-old children, and adults were asked to perform the DCCS task while their brain activation was examined with a multichannel NIRS system that covered the inferior prefrontal regions corresponding to F7/8 in the International 10/20 system. Brain activation during the preswitch and postswitch was separately analyzed, and compared to the activation during the control phases.

At the behavioral level, 5-year-old children and adult participants easily performed both the preswitch and postswitch phases (Moriguchi and Hiraki, 2009). Some 3-year-old children performed the DCCS tasks perfectly, but others committed perseverative errors during the postswitch phases. At the neural level, adults and 5-year-old children showed significant activation in the right and left inferior prefrontal areas during the preswitch and postswitch phases compared to the control phase. The researchers analyzed the 3-year-old children separately according to whether they committed perseverative errors during the tasks. In the children who performed perfectly (pass group), the right inferior prefrontal areas were significantly activated during the preswitch and postswitch phases. In contrast, children who perseverated (perseverate group) exhibited no significant activation in the inferior prefrontal areas during both the preswitch and postswitch phases.

The results suggest that the development of cognitive shifting was correlated with the activations in the prefrontal regions. However, it has been shown that the NIRS signal is the product of the optical path length and the hemoglobin changes, and the optical path length differs across participants and head positions (Zhao et al., 2002). Thus, the comparison or integration of data between different subjects may be difficult. Rather, research of within-subject designs can be appropriate to compare across different conditions within the subjects. Thus, longitudinal method may be useful to address the age-related changes in the activations in specific brain regions.

Moriguchi and Hiraki (2011) longitudinally examined the development of prefrontal activation in children. Children were given the DCCS task, and developmental changes in prefrontal activation were examined at 3 (Time 1) and 4 years of age (Time 2). Behavioral results indicated that children in the perseverate group (i.e., the children who committed errors at Time 1) improved their performances significantly. Children in the pass group (i.e., the children who did not commit errors at Time 1) performed correctly at Time 2. Thus, there were no significant behavioral differences between the children in the pass and perseverate groups at Time 2.

**Figure 2:**
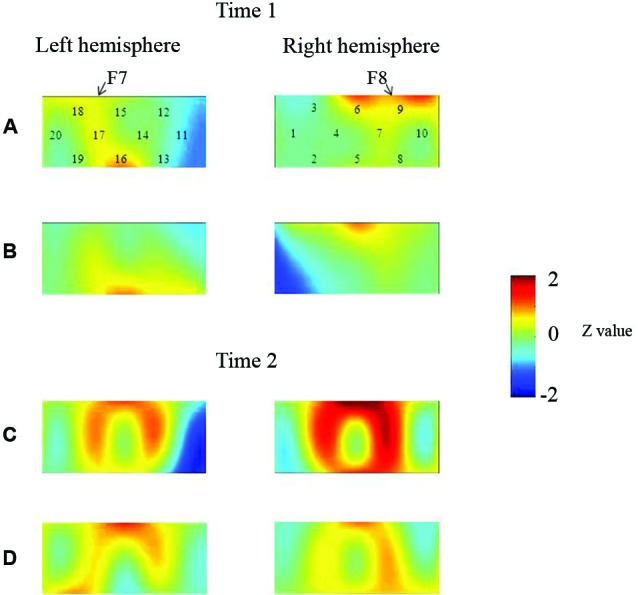
**The brain activations of children in the pass group at Time 1 (AB) and Time 2 (CD)**. The grand averaged data during the task phases are shown. The numbers (1–10) indicate the channel of the NIRS probe. **(A)** and **(C)** show the brain activations during the preswitch phases, and **(B)** and **(D)** show the brain activations during the postswitch phases. The red areas highlight areas where stronger activations were observed during the task, and the blue areas highlight areas where deactivations were observed during the task. Figure from Moriguchi and Hiraki (2011). Longitudinal development of prefrontal function during early childhood. *Developmental Cognitive Neuroscience*, 1, 153–162., with permission from Elsevier.

**Figure 3:**
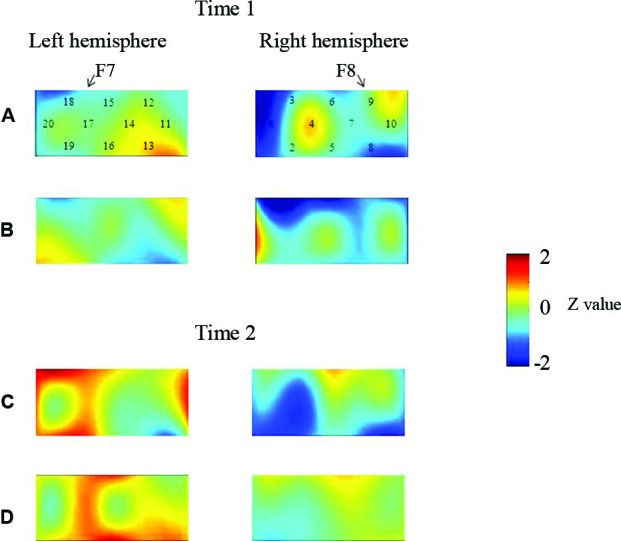
**The brain activations of children in the perseverate group at Time 1 (AB) and Time 2 (CD)**. The grand averaged data during the task phases are shown. The numbers (1–10) indicate the channel of the NIRS probe. **(A)** and **(C)** show the brain activations during the preswitch phases, and **(B)** and **(D)** show the brain activations during the postswitch phases. The red areas highlight areas where stronger activations were observed during the task, and the blue areas highlight areas where deactivations were observed during the task. Figure from Moriguchi and Hiraki (2011). Longitudinal development of prefrontal function during early childhood. *Developmental Cognitive Neuroscience*, 1, 153–162., with permission from Elsevier.

At the neural level, children in the pass group showed significant right inferior prefrontal activation during the preswitch and postswitch phases at Time 1 and Time 2, and had significant activity in the left inferior prefrontal areas during the preswitch and postswitch phases at Time 2 compared to Time 1 (Figure 2). The activation pattern at Time 2 was similar to that found in 5-year-old children in the study by Moriguchi and Hiraki (2009), with the children exhibiting bilateral inferior prefrontal activation during the DCCS task. On the other hand, the results for the perseverate group showed a different pattern (Figure 3; Moriguchi and Hiraki, 2011). At Time 1, children in the perseverate group exhibited no significant activation in the inferior prefrontal areas during the preswitch and postswitch phases whereas at Time 2, they showed significant activation in the left (but not right) inferior prefrontal regions during both phases.

Results of two studies showed that sustained unilateral (either right or left) inferior prefrontal activation across the preswitch and postswitch phases may be important for successful performance in the DCCS task. Similar results were obtained in an event-related potential study of DCCS (Espinet et al., 2012). Furthermore, there might be individual differences in the development of prefrontal function during preschool ages (Moriguchi and Hiraki, 2011). Children in the pass group showed activation of the right prefrontal regions at Time 1 and then recruited bilateral inferior prefrontal regions at Time 2. Children in the perseverate group showed no significant activation in the prefrontal regions at Time 1, but recruited the left inferior prefrontal regions at Time 2 when they passed the DCCS tasks. It should be noted that 3-year-old children in the pass group (who successfully performed the DCCS earlier) recruited the right inferior prefrontal areas, whereas children in the perseverate group (who successfully performed the task 1 year later than those in the pass group) recruited the left prefrontal regions. These results suggest that the right inferior prefrontal areas may be relatively dominant in DCCS tasks, while the left inferior prefrontal areas may support or compensate for right inferior prefrontal activations (Moriguchi and Hiraki, in press).

Few NIRS studies have been conducted on the development of inhibitory control in young children. Recently, Mehnert et al. (2013) gave 4- to 6-year-old children and adults a Go/NoGo task, where participants were asked to respond to targets by pressing a button (Go trials) and to avoid making a response to non-targets (NoGo trials). The researchers measured activity in several brain regions including the prefrontal, parietal, and temporal regions using NIRS. Behavioral results showed that adults performed the tasks more accurately and faster than children did. NIRS results showed that adults activated right frontal and parietal regions during NoGo trials compared to Go trials, whereas children’s right frontal and parietal activation was high in both Go and NoGo trials. Moreover, functional connectivity analyses revealed a stronger partial coherence in short-range connectivity in the right frontal and right parietal cortices in children compared to adults. In contrast, adults showed long-range functional connectivity between bilateral frontal and parietal areas. Although the research relied on concentration changes in deoxygenated hemoglobin, their results revealed that children activated the right frontal and parietal areas in the Go/NoGo task.

## Neural basis of working memory in young children

It has been repeatedly shown that regions in the prefrontal cortex, such as the dorsolateral prefrontal cortex, play an important role in visuospatial working memory in older children and adults as well as in non-human primates (Goldman-Rakic, 1995; Braver et al., 1997; Casey et al., 2005). However, little is known about the neural basis of working memory in young children.

Using NIRS, Tsujimoto et al. (2004) reported that the neural basis of working memory in young children covers the lateral prefrontal regions corresponding to Brodmann areas 9/46. In this study, 5- and 6-year-old children and adults performed a visuospatial working memory task. In this task, participants had to keep the locations of a sample cue array in mind during a delay period, after which they were asked to report whether a test cue location was identical to any of the sample cue locations. In adult participants, two (LOW condition) or four (HIGH condition) location cues were given as sample cue arrays, whereas children were given only two location cues. The authors examined prefrontal activity after presentation of the sample cues.

At the behavioral level, the adults’ performances in the HIGH condition were significantly worse than their performances in the LOW condition. Children’s performances were worse than adults’ performances both in the LOW and HIGH conditions. At the neural level, adult participants showed significant activation of the bilateral lateral prefrontal regions in the HIGH condition, whereas relatively weaker activation was observed in the LOW condition. These results suggest that memory load affected activity in the lateral prefrontal cortex. The brain regions and time course of activity in children were similar to those in adults, as children also exhibited sustained lateral prefrontal activation after onset of the sample cues. Tsujimoto et al. (2004) concluded that the lateral prefrontal cortex was activated in young children during a working memory process.

Tsujii et al. (2009) examined the longitudinal development of prefrontal function using the same visuospatial working memory task. Children participated in the study at 5 (Study 1) and 7 years of age (Study 2). A multichannel NIRS shell was placed on the prefrontal regions corresponding to Fp1/2 in the International 10/20 system. Behavioral results showed that children significantly improved their performance on the working memory task between Study 1 and Study 2. At the neural level, children exhibited bilateral prefrontal activation during the working memory task in both Study 1 and Study 2, although activity at age 7 (Study 2) was weaker than that at age 5 (Study 1). Importantly, in Study 2, the children exhibited laterality effects, with activation in the right prefrontal regions being stronger than that in the left prefrontal regions. Such laterality effects were not observed in Study 1. Tsujii et al. (2009) interpreted these findings as suggesting that visuospatial working memory induces right-lateralization, whereas verbal working memory induces left-lateralization.

Other researchers focused on the limits of working memory capacities (Buss et al., in press). It is well known that visual working memory can hold 3–4 items at any given moment (Vogel and Machizawa, 2004). These capacity limits are often indexed by the change detection task. The basic procedure is similar to that of the working memory tasks cited above. In this task, participants are shown a cue array and instructed to keep the array in mind during a delay phase. Then, participants are shown a test array where either all items are the same as the cue array, or some of the features are changed. Then, they are asked to report whether there were changes in the test array or not. Task difficulty depends on the number of items presented in the cue array. In research using children, the number of items is 1, 2, or 3.

Buss et al. (in press) gave 3- and 4-year-old children this task, and examined the neural activation after presentation of the cue array with an NIRS system that covered the prefrontal regions corresponding to F3–5/4–6 and the parietal regions corresponding to P3–5/4–6 in the International 10/20 system. Their results showed that children’s behavioral performances were a function of the number of items presented in the cue array. They performed worse when three items were presented than when one item was presented. The age effects were also significant, showing that 4-year-old children overall outperformed 3-year-old children. At the neural level, children exhibited significant activation in the frontal and parietal regions after presentation of the cue array. In addition, the activation was affected by the number of items. That is, activity in the left frontal areas and bilateral parietal areas was significantly stronger when three items were presented than when one or two items were presented. The age differences were evident in the parietal cortex, since stronger activation in this area was observed in 4-year-old children compared to 3-year-old children. In the right frontal cortex, activity in 3-year-old children first increased and then decreased. No such activation pattern was found in 4-year-old children. Interpretation of these results was difficult, but given that the activation patterns in the right prefrontal cortex were significantly correlated with behavioral performance, the right frontal regions may play an important role in visuospatial working memory.

Overall, research has consistently shown that children activate the right prefrontal regions during visuospatial working memory tasks. The developmental changes, however, were less clear. Some studies showed stronger activation in older children whereas other research showed weaker activation in older children. The mixed results may be due to the variation in task demands across studies. Generally, greater demand may induce stronger neural activation in the prefrontal cortex. Thus, it is possible that children exhibited age-related improvement in prefrontal activation as long as the task demands were appropriate. But in tasks that were too easy for older children, the older children might have had weaker prefrontal activation compared to younger children.

## EF in children with autism

Neuroimaging research with NIRS might also contribute to our understanding of developmental disorders, such as autism spectrum disorder (ASD). This disorder is characterized by deficits in social interaction and communication, as well as restricted, repeated, and stereotyped interests and behaviors (American Psychiatric Association, 2000). There are several cognitive theories for explaining the deficits of ASD, but one theory that might be related to stereotyped behavior involves EF (Hill, 2004). Some previous studies have revealed that patients with ASD have difficulties with cognitive shifting during the WCST (Ozonoff et al., 1994), but other research did not support the results (Nydén et al., 1999). The mixed results may be due to that WCST task includes many complex cognitive processes, such as planning, cognitive shifting, response inhibition, and error detection. Moreover, few brain imaging studies have investigated young children with ASD. Thus, it was unclear whether children with ASD may have functional and anatomical deficits in the prefrontal cortex.

Recently, some studies using NIRS have indicated behavioral and neural differences in cognitive shifting between typically developing (TD) children and children with ASD (Yasumura et al., 2012). Seven- to 12-year-old ASD children and aged-matched TD children were asked to perform an advanced version of DCCS tasks (ADCCS), while neural activity in the prefrontal cortex including F7/F8 was examined with NIRS. In the ADCCS, children need to switch flexibly between two incompatible rules within the same set. Half of the test cards have a border around them, while the other half do not. Children are asked to sort the cards according to one rule if the card has a border and according to another rule if the card has no border. Children typically have more difficulty performing the ADCCS task than the standard DCCS task (Hongwanishkul et al., 2005; Moriguchi and Hiraki, in press). The behavioral results revealed that children with ASD performed the ADCCS significantly worse than TD children did. The NIRS results demonstrated significant differences in prefrontal activation between the groups. TD children exhibited significant bilateral prefrontal activation during the ADCCS. In contrast, children with ASD showed significant left prefrontal activation, but the right prefrontal regions were not significantly activated. A direct comparison between the groups revealed significant differences in right prefrontal regions.

Xiao et al. (2012) examined the neural basis of inhibitory control in children with autism and attention-deficit/hyperactivity disorder (ADHD) using NIRS. In this study, TD children, children with high functioning autism (HFA) and children with ADHD were given color-word Stroop and Go/NoGo tasks. The research examined the activations in the forehead. As the results, there were no significant behavioral differences and the prefrontal activations between groups during the Stroop task. On the other hand, in the Go/NoGo task, children with autism and ADHD made more commission error during the NoGo blocks than typically developed children, and children with autism and ADHD did not differ in the errors. Moreover, the NIRS results showed that children with ASD and children with ADHD exhibited weaker right prefrontal activations during the Nogo block than TD children. The different results in the Stroop and Go/NoGo tasks may be due to that different brain regions may be recruited in each task. Indeed, it has been shown that Go/NoGo tasks mainly activate the right prefrontal regions (Aron et al., 2004) whereas the Stoop tasks recruit anterior cingulate regions (Pardo et al., 1990). The results in Stroop tasks were discussed later.

Taken together, although the evidence is not enough, these results show that children with ASD may have some difficulty with cognitive shifting and inhibitory control at both the behavioral and neural level.

## EF in children with attention-deficit/hyperactivity disorder

Executive dysfunction may be related to ADHD (Barkley, 1997). This developmental disorder is characterized by inattention, hyperactivity, and impulsivity (American Psychiatric Association, 2000). Recent neuroanatomical research suggests that children with ADHD exhibit a marked delay in maturation of the prefrontal areas (Shaw et al., 2007). Moreover, it has been shown that patients with ADHD exhibit weaker prefrontal activation in EF tasks (Pliszka et al., 2006). These studies indicate that patients with ADHD may have functional and anatomical deficits in the prefrontal cortex (Bush et al., 2005). However, few brain imaging studies have investigated young children with ADHD.

Yasumura et al. (in press) asked school-aged children with ADHD, children with ASD and TD children to perform coro-word Stroop and reverse Stroop tasks. In the Stroop task, participants had to select a color word (e.g., red) from among four color words shown in each corner of the screen that matched the word displayed in the center of the screen. The central word was the name of a color displayed in an incongruent font color (e.g., the word “green” displayed in red font). Participants had to choose the word that matched the font color of the central word and inhibit the tendency to select the word that matched the meaning of the central word. In the reverse Stroop task, participants had to select a color from among four colored patches shown at each corner of the screen that matched the meaning of the central word. The font color was incongruent with the central word meaning and interfered with the choice of matching the word with the correct colored patch. Participants had to inhibit the tendency to select the color that matched the font color of the central word. It has been shown that the reverse Stroop task may induce participants’ conflict although the conflict may be smaller than that in the standard Stroop task (Ruff et al., 2001).

Behavioral results revealed that there were no significant differences between the groups on the Stroop task. In the reverse Stroop task, children with ADHD committed more errors than those with ASD and TD children. During the tasks, NIRS was used to examine activity in the prefrontal regions corresponding to F7/8 and FpZ in the International 10/20 system. Consistent with the behavioral data, no significant differences between groups were found for the Stroop task. On the other hand, in the reverse Stroop task, children with ADHD exhibited significantly lower activation in the right prefrontal regions than TD children did. The results revealed that children with ADHD exhibited abnormal behavioral performance and neural activation in the right prefrontal regions. Interestingly, children with ASD did not show such abnormalities.

The results of Stroop tasks were mixed. As noted above, Xiao et al. (2012) reported that there were no significant behavioral differences and the prefrontal activations between TD children and children with ADHD during the Stroop task. On the other hand, Negoro et al. (2010) reported that TD children and children with ADHD differed in changes of the oxygenated hemoglobin in the bilateral prefrontal regions during the color-word Stroop task. The different results may be due to the differences in task design. Yasumura et al. (in press) asked children to choose the correct words. Xial et al. (2012) also asked children to manually respond to the stimuli. On the other hand, in Negoro et al.’s (2010) study, children verbally responded the words during the Stroop task. It has been shown that NIRS signals on forehead were strongly affected by skin blood flow in a verbal fluency task (Takahashi et al., 2011). Given that, it is possible that the activations in the prefrontal regions were strongly affected by skin blood flow in the verbal Stroop task. The signal changes in the verbal task were relatively large compared to the manual task, by which researchers may easily detect the differences between groups.

In another study, Tsujimoto et al. (2013) examined whether children with ADHD showed deficits in visuospatial working memory. The basic procedure of the task was the same as the procedure mentioned above for Tsujimoto et al. (2004). Briefly, participants had to keep the locations of a sample cue array in mind during a delay period. However, in this study, there were two conditions after the delay period. In the distractor condition, participants were given a distractor task, where three purple dots and three yellow dots appeared at random locations on a screen, and the participants had to touch only the yellow dots. In the no-distractor condition, there were no distractor tasks. After that, white dots appeared on the screen, and participants had to touch the positions where sample cues had been presented.

Using NIRS, activity in the prefrontal regions corresponding to F7/8 and FpZ in the International 10/20 system of school-aged ADHD children and TD children was examined while they performed the working memory task. Children with ADHD showed poorer performance on the working memory tasks than TD children. Specifically, children with ADHD made more errors in the distractor condition than in the no-distractor condition, but no such differences were found in TD children. NIRS results revealed significant differences in prefrontal activation between children with and without ADHD in the distractor condition. Specifically, stronger activation in the right and middle prefrontal regions was observed for children with ADHD than for those without ADHD. No such differences were found in the no-distractor condition. Moreover, a significant correlation between error rates and right prefrontal activation was found in children with ADHD. Thus, hyperactivity in the right prefrontal regions may play a role in error-making during working memory tasks (Tsujimoto et al., 2013).

The research shows that children with ADHD may have some difficulty with inhibitory control indexed by Go/NoGo and working memory at both the behavioral and neural level. With regards to the Stroop tasks, the results were mixed. It should be noted that the activation in the prefrontal regions was sometimes stronger and sometimes weaker in children with ADHD that in TD children. Tsujimoto et al. (2013) suggested that hyperactivity observed in ADHD children may be due to the compensation of the inefficient neural processing in the prefrontal regions. Thus, the children may recruit the prefrontal regions in the EF tasks, but the activations may not be efficient enough to perform the tasks successfully. On the other hand, the hypoactivations observed children with ADHD may be due to that they fail to recruit the prefrontal regions in the EF tasks. As young children generally failed to activate the prefrontal regions, children with ADHD may fail to activate the prefrontal regions. Nevertheless, there is little data regarding the issue. Future research should be done to address it.

## Methodological issues

We had to note that there were several methodological issues in NIRS research for young children. First, we reviewed previous research focusing on oxygenated hemoglobin because most of the previous research on young children consistently reported the oxygenated hemoglobin as an index of brain activation. It has been shown that BOLD signal showed significant correlations with the oxygenated hemoglobin measured by NIRS (Strangman et al., 2002). A recent study using a working memory task and a finger tapping task revealed that oxygenated (and deoxygenated) hemoglobin were significantly correlated with BOLD signals in the prefrontal and the sensorimotor regions (Sato et al., 2013). The results indicate that the change in oxygenated hemoglobin is good indicator of brain activity in EF studies.

However, there are reasons why researchers should report both oxygenated and deoxygenated hemoglobin changes. The NIRS research is partly based on evidence of other neuroimaging technique such as fMRI, and it is well known that BOLD signal in fMRI tightly correlates, and have common physiological origins, with the deoxygenated hemoglobin (Huppert et al., 2006). There were mixed evidence regarding whether either oxygenated or deoxygenated hemoglobin showed the better correlation with BOLD signals. Huppert et al. (2006) argued that the mixed results may be due to low signal-to-noise ratio in NIRS measurements, and showed that, with high signal-to-noise ratio in the measurements, deoxygenated hemoglobin was better correlated with BOLD signals than oxygenated hemoglobin in a short-duration motor task. Further, it has been shown that body motions can cause baseline fluctuation in the NIRS signal (Yamada et al., 2009). Importantly, oxygenated hemoglobin is more highly influenced by the motions than deoxygenated hemoglobin. Such fluctuations can be removed by task designs and separating signal components, but some of the research did not consider such removals. Given the evidence, we suggest that both oxygenated and deoxygenated hemoglobin changes should be analyzed and reported.

Second, the spatial and depth sensitivity in NIRS measurement should be considered. The sensitivities to the brain tissues are dependent on the placement of emitters and detectors. Here, we focused on the depth sensitivity. NIRS system examines brain activation in the upper areas of the cerebral cortex. This is because of the fact that the near-infrared light is predominantly absorbed by the brain tissue hemoglobin at approximately 10–30 mm below the scalp. Simultaneous measurement studies have shown that the best correlation between the NIRS signal and parameters in other imaging method, such as PET (Hock et al., 1997) and fMRI (Schroeter et al., 2006), was at a depth of approximately 1–1.5 cm from the skin. Nevertheless, more recent research has shown that the depth sensitivity at a depth of around 1 cm into the intracranial space is quite low by the thickness of several overlying tissue layers, such as scalp (Strangman et al., 2013). Although there is little evidence regarding this issue in young children, we had to note that the NIRS system cannot measure the activations of the deeper areas in the brain.

Third, some of the previous study used between subject designs, which may have a problem. As noted above, the direct comparison or integration of data between subjects is difficult because the optical path length differs cross participants and head positions (Zhao et al., 2002). Thus, longitudinal or microgenetic method may be appropriate to address the developmental changes in activations of specific brain regions. Moreover, even in the longitudinal study, researchers had to care about the comparisons between several different time points. Brain as well as superficial tissues such as scalp and skull are developing during childhood. A MRI study showed that there were developmental changes in the distance between scalp and brain in children. The distance depends on children’s age and the brain regions (Beauchamp et al., 2011), but the frontal regions showed age-related changes in the distance until middle childhood. Coupled with the issue of the depth above, we have to consider such data to analyze and interpret the results in the NIRS signals.

Despite the limitations, NIRS has several advantages for research on infants and young children. For example, as noted above, NIRS is noninvasive and does not require very exact fixations of body and head such that other neuroimaging methods require. Children can sit in a chair during an experiment. Also, a NIRS experiment can be conducted silently compared to an fMRI experiment. The facts make research on infants and young children easier. Nevertheless, there would be motion artifacts in young children’s research, which may benefit from analyses that separated functional brain activities from other components in NIRS signals (Scholkmann et al., in press). In addition, the NIRS is portable and less costly. We can measure children’s brain activations in natural settings, such as children’s home, kindergartens or nursery schools as well as an experimental room. Moreover, NIRS can apply to children with developmental disorders and behavioral difficulties, and have the potential for the use of their interventions.

## Conclusion and future directions

Collectively, the results of these studies show that both children and adult participants show significant activation in the prefrontal regions when performing cognitive shifting, inhibitory control and working memory tasks. Importantly, the children with ADHD and those with ASD who had difficulties with the tasks exhibited abnormal activation in the prefrontal areas. Taken together, the studies discussed in this review suggests that activation in the prefrontal regions may be important for successful performance on EF tasks in young children.

The next step is to determine how the development of prefrontal function may be related to other aspects of cognitive and social development. It has been shown that the development of EF correlates with the development of socio-cognitive skills, such as theory of mind, communicative skills, and emotional regulation (Dempster, 1992; Eisenberg et al., 1997; Carlson and Moses, 2001; Moriguchi et al., 2008). Given the correlational evidence, researchers have suggested that EF may contribute to the emergence of such skills (Moses, 2005). However, the exact mechanisms of the relationship between socio-cognitive skills and EF are still unclear. NIRS may aid in understanding such relationships. In fact, recent research has examined the relationship between prefrontal activation and emotion regulation (Fekete et al., in press; Perlman et al., in press). However, further research should be conducted to examine the exact mechanism underlying this relationship.

Another issue is how prefrontal activation may change developmentally across different tasks (e.g., working memory and cognitive shifting tasks). On the behavioral level, a single-factor model (general EF) is sufficient to account for the data for preschool-aged children (Wiebe et al., 2008). The available results suggest the possibility that the prefrontal regions may be generally activated across different tasks in younger children, but may become localized to specific regions in older children. Johnson (2011) proposed that some regions in the cerebral cortex may start with broad functionality, and consequently are partially activated in different stimuli and tasks. Indeed, Durston et al. (2006) reported the developmental shift from diffuse to focal activations in the prefrontal regions when school-aged children were given a Go/NoGo task.

In addition, other brain regions may be activated during these tasks. Given the limitation of the NIRS system, this review focused on the role of the prefrontal regions in the development of EF. However, using fMRI, Morton et al. (2009) reported that school-aged children showed significant activation of the superior parietal cortex, dorsolateral prefrontal cortex, and presupplementary motor regions during the DCCS task. Furthermore, Monchi et al. (2001) have revealed that the dorsolateral prefrontal cortex and parietal cortex are significantly activated in adults during the WCST. Thus, future studies should examine activation in other brain regions in young children during EF tasks. Some recent reports examined other brain regions as well as the prefrontal regions during EF tasks in young children (Buss et al., in press). Such examinations may lead to a better understanding of the brain mechanisms involved in the development of EF in young children.

## Conflict of interest statement

The authors declare that the research was conducted in the absence of any commercial or financial relationships that could be construed as a potential conflict of interest.
